# Bio-Converted Spirulina for Nutraceutical Chewing Candy Formulations Rich in L-Glutamic and Gamma-Aminobutyric Acids

**DOI:** 10.3390/microorganisms11020441

**Published:** 2023-02-09

**Authors:** Elena Bartkiene, Ernesta Tolpeznikaite, Dovile Klupsaite, Vytaute Starkute, Vadims Bartkevics, Anna Skrastina, Romans Pavlenko, Ernestas Mockus, Vita Lele, Gabija Batkeviciute, Ausrine Budrikyte, Rusne Janulyte, Ieva Jomantaite, Auguste Kybartaite, Karolina Knystautaite, Aiste Valionyte, Romas Ruibys, João Miguel Rocha

**Affiliations:** 1 Institute of Animal Rearing Technologies, Faculty of Animal Sciences, Lithuanian University of Health Sciences, 44307 Kaunas, Lithuania; 2 Department of Food Safety and Quality, Faculty of Veterinary, Lithuanian University of Health Sciences, 44307 Kaunas, Lithuania; 3 Institute of Food Safety, Animal Health and Environment “BIOR”, Zemgales Priekšpilsēta, LV-1076 Riga, Latvia; 4 Institute of Agricultural and Food Sciences, Agriculture Academy, Vytautas Magnus University, 44307 Kaunas, Lithuania; 5 LEPABE-Laboratory for Process Engineering, Environment, Biotechnology and Energy, Department of Chemical Engineering (DEQ), Faculty of Engineering, University of Porto (FEUP), Rua Roberto Frias, s/n, 4200-465 Porto, Portugal; 6 ALiCE—Associate Laboratory in Chemical Engineering, Faculty of Engineering, University of Porto, Rua Dr. Roberto Frias, s/n, 4200-465 Porto, Portugal

**Keywords:** spirulina, nutraceuticals, fermentation, gamma-aminobutyric acid, L-glutamic acid, fatty acids, emotions

## Abstract

This study aimed at evaluating changes of microalgae Spirulina during its fermentation with *Lactiplantibacillus plantarum* No. 122 strain, and further at incorporating Spirulina bio-converted for nutraceuticals rich in L-glutamic (L-Glu) and gamma-aminobutyric acids (GABA) into sucrose-free chewing candy (gummy) preparations. Fermented Spirulina had higher b* (yellowness) coordinates than untreated (non-fermented), and fermentation duration (24 and 48 h) had a statistically significant effect on colour coordinates. The highest contents of L-glutamic and gamma-aminobutyric acids (4062 and 228.6 mg/kg, respectively) were found in 24 and 48 h-fermented Spirulina, respectively. Fermentation increased the content of saturated fatty acids and omega-3 in Spirulina, while monounsaturated fatty acids and omega-6 were reduced. The addition of fermented Spirulina (FSp) significantly affected hardness, decreased lightness and yellowness, and increased the greenness of chewing candies. All chewing candy samples (with xylitol) prepared with 3 and 5 g of FSp and 0.2 µL of *Citrus paradise* essential oil received the highest scores for overall acceptability, and the highest intensity (0.052) of emotion “happy” was elicited by the sample group containing xylitol, agar, ascorbic acid, 3 g of FSp, and 0.1 µL of *Mentha spicata* essential oil. As an outcome of this research, one may conclude that fermented Spirulina has significant potential as an innovative ingredient in the production of healthier sucrose-free nutraceutical chewing candies.

## 1. Introduction

Value-added functional foods are a relatively new category of products. This concept started in Japan in 1984 [1], since then the market of functional foods has grown consistently [2]. Spirulina is a valuable material for the preparation of functional products because of a large number of health-promoting benefits. It was reported that Spirulina has therapeutic applications in many non-communicable diseases [3]. Nowadays, higher than 30% of world microalgal production is from Spirulina [4]. In addition to functional constituents (chlorophylls, carotenoids, phycobiliproteins, etc.), Spirulina is mainly known because of its high protein content (60–70% dry matter), which is related to biomass growth conditions [5,6]. Most of the produced Spirulina is consumed as an added-value supplement (“superfood”) and sold in various forms—chiefly, powder, flakes, and capsules [7]. Because of its health benefits and high potential to become an important ingredient in the creation of innovative functional foods, the development of various forms of nutraceuticals, including chewing candies, has become very attractive. Spirulina has a high content of essential amino acids based on the Food and Agriculture Organization (FAO) of the United Nations and World Health Organization (WHO)’s conception of an “ideal” protein [8]. The most valuable proteins in Spirulina are phycocyanin, allophycocyanin, and phycoerythrin.

During its consumption, in vivo, microalgae interact with gastrointestinal tract microorganisms [9,10]. This interaction can lead to the production of functional molecules, including neurotransmitters such as gamma-aminobutyric acid (GABA) [9,11]. The latter is a non-protein amino acid widely distributed in nature [12]. Experimental evidence has shown many benefits of these molecules on brain neuronal survival [13,14,15,16,17,18,19]. Moreover, the main non-essential amino acid in Spirulina and Spirulina protein isolates is glutamic acid (9.57 ± 0.27 g/100 g) [20], and microorganisms can metabolise this amino acid to GABA [12].

Many bacteria, including lactic acid bacteria (LAB) [21], are able to synthesise GABA. In this study, we hypothesised that fermentation with *Lactiplantibacillus plantarum* No. 122 strain can lead to an increment in Spirulina value as an ingredient for nutraceutical formulation, including with respect to the formation of GABA and their precursor, glutamic acid. Additionally, to increase the health benefits of the developed products, sucrose was changed to xylitol in this study.

It is well known that sucrose can result in weight gain and type-2 diabetes because of its highly metabolic activity [22]. Xylitol sweetness level is comparable to saccharose but its energy value is only 2.4 kcal/g; it dissolves well in water and demonstrates stability at high temperatures (i.e., it does not caramelise during heating) [23,24,25,26,27]. Xylitol is often used in pharmaceutical products, but it is also a natural compound found in some fruits and vegetables [28]. Additionally, xylitol promotes the growth of beneficial gut microbiota [27,29]. The use of xylitol 3–5 times every day may help increase bone mass and improve health; however, the total intake should not exceed 10 g [30]. Moreover, it should be mentioned that the consumption of polyols in excess of 50 g/day may cause persistent diarrhoea [28]. Finally, taking into consideration that nutraceuticals in the form of chewing candy will not be used in very high contents, xylitol is an appropriate ingredient to increase their overall sensory acceptability by providing sweet taste.

Another challenge for the products containing Spirulina is their specific odour, which is typical for microalgae. To mask this odour, two essential oils were tested in this current study: *Citrus paradise* and *Mentha spicata*. The main compounds of Citrus essential oils are monoterpene hydrocarbons and limonene [31]. The latter possess strong antioxidant and radical scavenging activities [32], and may be the key factor to the anti-inflammatory activity of Citrus essential oils [33]. Spearmint essential oil includes carvone, carveol, dihydrocarvone, dihydrocarveol, and dihydrocarvyl acetate [34]. Regarding this latter essential oil, its antimicrobial, antioxidant, insecticidal, antitumor, anti-inflammatory, and antidiabetic activities have been reported [34].

Based on the above, the aim of this study was to evaluate the changes of Spirulina during fermentation with *Lactiplantibacillus plantarum* No. 122 strain, and further to incorporate Spirulina bio-converted for nutraceuticals rich in L-glutamic (L-Glu) and GABA acids into sucrose-free chewing candy preparations.

## 2. Materials and Methods

### 2.1. Principle Scheme of the Experiment

**Figure 1:**
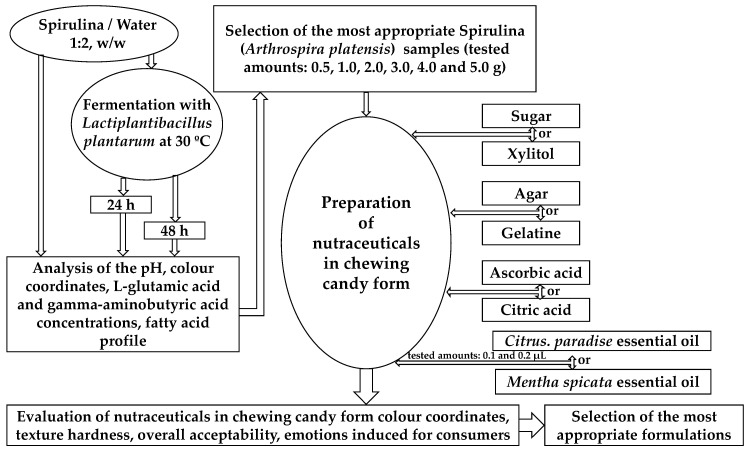
Principle scheme of the experiment.

The principle scheme of the whole experiment is shown in Figure 1. In the first stage of the experiment, Spirulina samples were fermented and their parameters (pH, colour coordinates, L-Glu and GABA concentrations, and fatty acid profile) were evaluated. In the second stage, for the nutraceuticals in the form of chewing candy preparations, the most appropriate fermentation duration (24 and 28 h) of Spirulina was selected, and different quantities of Spirulina (0.5, 1.0, 2.0, 3.0, 4.0, and 5.0 g) were tested during nutraceutical preparations. Additionally, different sweet tasting (sucrose and xylitol), texture forming (agar and gelatine), sour tasting (ascorbic acid and citric acid), and Spirulina odour masking (*Citrus paradise* and *Mentha spicata* essential oils) ingredients for the nutraceutical formulations were tested. The nutraceutical chewing candies were subjected to analyses of colour coordinates, texture hardness, and overall acceptability. Additionally, samples showing the highest acceptability were tested by using the face reading technique, which indicates the intensity of the emotions induced by the tested samples in the panellists of the trained tasting panel.

### 2.2. Lactic Acid Bacteria (LAB) and Spirulina Used in the Experiment

The LAB strain *Lactiplantibacillus plantarum* No. 122 was acquired from the Lithuanian University of Health Sciences collection (Kaunas, Lithuania). Before the experiment, *Lactiplantibacillus plantarum* No. 122 strain was incubated and multiplied in a De Man, Rogosa, and Sharpe (MRS) broth culture medium (Biolife, Milano, Italy) at 30 °C under anaerobic conditions for 24 h.

Lyophilised Spirulina (*Arthrospira platensis*) powder (content per 100 g: 1.1 g of sodium, 30.3 g of total carbohydrates, 60.6 g of proteins, 151.5 mg of calcium, 1.7 mg of potassium, and 48.5 mg of iron) was provided by Now Foods Company (Bloomingdale, IL, USA).

A total of 3 mL of fresh *Lactiplantibacillus plantarum* No. 122 strain grown on MRS broth (average cell concentration of 9.0 log_10_ CFU/mL) was inoculated in 100 mL of Spirulina/water mixture (Spirulina/water ratio of 1:2, *w*/*w*), and fermented at 30 °C under anaerobic conditions for 24 and 48 h.

### 2.3. Analysis of pH and Colour Coordinates (L*, a* and b*) in Spirulina Samples

The pH of Spirulina/water mixture was evaluated with a pH meter (Inolab 3, Hanna Instruments, Venet, Italy) by inserting the pH electrode into the samples. The colour coordinates of the Spirulina/water mixture were evaluated on the samples surface using the International Commission on Illumination (CIE) L*a*b* colour space system (CromaMeter CR-400, Konica Minolta, Marunouchi, Tokyo, Japan) [35].

### 2.4. Evaluation of L-Glutamic Acid (L-Glu) and Gamma-Aminobutyric Acid (GABA) Concentration in Spirulina/Water Mixture Samples

Half a gram of Spirulina/water mixture samples was extracted in 50 mL of Milli-Q water for 10 min using an overhead shaker. Samples were incubated for 30 min at 60 °C in a water bath. Then the tubes were cooled down and centrifuged at 4500 rpm for 10 min. A 1 mL aliquot of the supernatant was transferred into 15 mL polypropylene test tubes and diluted with 9 mL of Milli-Q water (dilution 10×). Finally, samples were filtered and transferred to a 2 mL auto-sampler vial. Analyses were performed on a TSQ Quantiva MS/MS coupled to a Thermo Scientific Ultimate 3000 HPLC instrument (Thermo Scientific, Waltham, MA, USA). Chromatographic separation was carried out on a Luna Omega Polar C18 (2.1 mm diameter × 100 mm length, 3.0 μm-ϕ particle size) column at 40 °C using an injection volume of 5 μL. The mobile phase consisted of a 0.5 mM ammonium acetate solution in Milli-Q water (eluent A), and methanol (eluent B). A flowrate of 0.2 mL/min was used. The following gradient conditions were employed: 0.00 min, 1% B (99% A); 1.00 min, 1.0% B (99% A); 6.00 min, 99% B (1% A); 7.50 min, 99% B (1% A); 8.00 min, 1% B (99% A); and 10.00 min, 1% B (99% A). LC–MS interface conditions for the ionization of gamma-aminobutyric acid and L-glutamic in the positive ESI mode were as follows: needle voltage + 4500 V; sheath gas 60 Arb; aux gas 25 Arb; sweep gas 5 Arb; ion transfer tube temperature 200 °C; vaporiser temperature 350 °C. The main fragments were identified using the selected reaction monitoring (SRM), with the following ionic transitions: GABA (m/z 104 > m/z 45.151, CE 25.72 V; m/z 104 > 69.165, CE 15.92 V; m/z 104 > m/z 87.36, CE 10.66 V); L-Glu (m/z 148 > m/z 56.05, CE 30 V; m/z 148 > m/z 84, CE 30 V). Method recovery ranged from 59 to 112% for GABA and from 58 to 152 % for L-Glu. Method repeatability ranged from 5 to 23% for GABA and from 1 to 20 % for L-Glu. The results were obtained in some rounds of experiments on different days.

### 2.5. Analysis of Fatty Acid (FA) Profile in Spirulina/Water Mixture Samples

The extraction of lipids for fatty acids (FA) analysis was conducted with chloroform/methanol (2:1, *v*/*v*), and fatty acid methyl esters (FAME) were prepared according to Pérez-Palacios et al. [36]. The FA composition in Spirulina/water mixture samples was identified using a gas chromatograph GC 2010 Plus (Shimadzu Europa GmbH, Duisburg, Germany) equipped with mass spectrometer GC-MS QP2010 (Shimadzu Europa GmbH, Duisburg, Germany). Separation was carried out on a Stabilwax-MS column (30 m length × 0.25 mm internal diameter, and 0.25 μm-ϕ particle size) (Restek Corporation, Bellefonte, US). Oven temperature program started at 50 °C, then increased at a rate of 8 °C/min to 220 °C, held for 1 min at 220 °C, increased again at a rate of 20 °C/min to 240 °C, and finally held throughout 10 min. The injector temperature was 240 °C, interface 240 °C, and ion source 240 °C. The carrier gas was helium at a flowrate of 0.91 mL/min. The individual FAME peaks were identified by comparing their retention times with FAME standards (Merck & Co., Inc., Kenilworth, NJ, USA).

### 2.6. Preparation and Analysis of Nutraceutical Chewing Candy Formulations

The polymer agar powder (algae *Gelidium sesquipedale*, Rapunzel, Germany) was used as texture forming with mucoadhesive properties for nutraceutical chewing candies. In addition, gelatine was also tested (Klingai, Lithuania). Xylitol (Natur Hurtig, Nuremberg, Germany), citric acid (Sanitex, Kaunas, Lithuania), and sugar (“Nordic Sugar Kėdainiai“, Kedainiai, Lithuania) were purchased in a local market (JSC ‘Maxima LT’, Kaunas, Lithuania). Ascorbic acid (JSC “Stada Baltics”, Vilnius, Lithuania) was purchased in a local pharmacy company (JSC “Eurovaistine, Kaunas, Lithuania), and grapefruit (*Citrus paradise*, producer JSC “Zolotonošskaja PKF”, Komunarovskaja, Ukraine) and mint (*Mentha spicata*, producer JSC “Naujoji Barmune“, Vilnius, Lithuania) essential oils were obtained from JSC “Gintarine vaistine” (Kaunas, Lithuania).

The formula of the control chewing candy (/gummi) group consisted of sugar (17 g), water (20 mL), citric acid (0.7 g) or ascorbic acid (0.9 g), and agar (4.6 g) or gelatine (8.5 g). Furthermore, in the formulation of gummies, sugar was changed by xylitol, and the basic recipe of chewing candy formulation consisted in xylitol (17 g), water (20 mL), citric acid (0.7 g) or ascorbic acid (0.9 g), and agar (4.6 g) or gelatine (8.5 g) (Table 1).

Nutraceutical chewing candies were prepared by including different quantities of fermented Spirulina, and mint and grapefruit essential oils were used as Spirulina odour masking agents. Nutraceuticals in chewing candy formulations are given in Table 1.

For the preparation of nutraceutical chewing candies, firstly, agar or gelatine powder was soaked in water for 30 min and afterwards melted by heating for 15 min at 90 °C. Sugar or xylitol was added and dissolved in the mixture under boiling. The obtained mixture was further heated to 90 °C under stirring. Citric acid or ascorbic acid and different quantities of fermented Spirulina and essential oils were incorporated into nutraceutical chewing candy mass at the end of the process (mass temperature 40 °C). The obtained mass after mixing was poured into a cast, and nutraceutical gummies were dried at 22 ± 2 °C for 24 h to obtain a gel-hard form.

The hardness of nutraceutical chewing candies was evaluated by Texture Profile Analysis (TPA) using a Texture Analyser TA.XT2 (StableMicro Systems Ltd., Godalming, UK) (compression force 0.5 N, test speed 0.5 mm/s, post-test speed 2 mm/s and distance 6 mm). Sensory analysis of nutraceuticals was carried out according to the ISO 6658 method [37]. Thirty panellists evaluated the overall acceptability (OA) of gummies using the hedonic scale from 0 (extremely dislike) to 10 (extremely like).

After obtaining an optimal Spirulina and essential oil content, according to overall acceptability, the most acceptable samples were further analysed by evaluating nutraceutical emotions induced in panellists using the FaceReader 6.0 software (Noldus Information Technology, Wageningen, The Netherlands) and scaling eight emotion patterns (neutral, happy, sad, angry, surprised, scared, disgusted, and contempt). The panellists were asked to rate the nutraceutical samples during and after consumption with an intentional facial expression, which was recorded and then characterised by FaceReader 6.0. The panellists were asked to taste the whole presented sample at once, take 15 s to reflect on the taste impressions, then give a signal with a hand and visualised the taste experience of the sample with a facial expression best representing their liking of the sample. The whole procedure was filmed using a high-resolution Microsoft LifeCam Studio webcam mounted on a laptop facing the participants and Media Recorder (Noldus Information Technology, Wageningen, The Netherlands) software. Special care was taken to ensure good illumination of participants’ faces. The recordings, using a resolution of 1280 × 720 at 30 frames per second, were saved as AVI files and subsequently analysed frame by frame with FaceReader 6.0 software. For each sample, the section of intentional facial expression (from the exact point at which the subject had finished raising their hand to give the signal until the subject started lowering their hand again) was extracted and used for statistical analysis.

### 2.7. Statistical Analysis

Fermentation of Spirulina samples was performed in duplicate and all analytical experiments were carried out in triplicate (n = 6). Preparation of the nutraceutical chewing candies was performed in duplicate and analysis of the colour coordinates and texture hardness were carried out in triplicate (n = 6). Overall acceptability of samples as well as emotions induced by testing nutraceuticals were evaluated by thirty panellists. The mean values were calculated using the statistical package IBM^®^ SPSS^®^ for Windows [v28.0.1.0 (142), SPSS, Chicago, IL, USA]. The data were compared using Duncan’s multiple range test with significance defined at *p* ≤ 0.05. A linear Pearson’s correlation was used to quantify the strength of the relationship between the variables. Results were recognised as statistically significant at *p* ≤ 0.05.

## 3. Results and Discussion

### 3.1. Parameters of Non-Treated and Fermented Spirulina Samples

The pH values and CIE colour coordinates (L*, a* and b*) of Spirulina samples are given in Table 2. Comparing lightness (L*) coordinates in non-treated and fermented Spirulina samples, the highest L* was showed in fermented samples for 24 h with *Lactiplantibacillus plantarum* No. 122 strain (24.5 NBS). L* coordinates of non-fermented and 48 h-fermented samples were, in comparison with 24 h-fermented samples, 8.37% lower on average. The lowest a* coordinates (redness) was obtained in 24 h-fermented samples; non-fermented and 48 h-fermented samples a* were, on average, 8.46 and 9.23 times lower, respectively. In all cases, fermented samples showed higher b* coordinates (yellowness), in comparison with non-treated samples (on average 25.1 and 11.4% lower, when comparing with 24 and 48 h-fermented samples, respectively). Fermented Spirulina pH was, on average, 4.80; in comparison with non-fermented samples, their pH was, on average, 24.2% lower. Negative moderate correlation between pH and b* coordinate values of samples was disclosed (r = −0.775, *p* = 0.014). In addition, fermentation duration was a statistically significant factor on the colour coordinates of Spirulina samples (*p* ≤ 0.0001).

The main carotenoids of Spirulina are astaxanthin, zeaxanthin, and β-carotene [2], and canthaxanthin and lutein are also found at lower concentrations [38]. Additionally, Spirulina contains chlorophyll [39]. During this study, it was revealed that non-fermented and after 48 h of fermentation, Spirulina samples show lower L* values in comparison with 24 h-fermented samples. It could be that cell lysis released the pigments; however, after 48 h fermentation, they were degraded in medium rich metabolites and under the action of low pH values. Similar tendencies of a* coordinates were encountered. The lowest b* coordinates were obtained in control samples. As can be seen in Table 2, after 24 h of fermentation, b* coordinates increased and were followed by a decrease after 48 h of fermentation.

Gamma-aminobutyric acid and L-Glu concentrations in Spirulina samples are shown in Table 3. In comparison with non-fermented samples, 24 h-fermented samples, on average, demonstrated 9.4 and 1.77 times higher GABA and L-Glu contents, respectively. After 48 h of fermentation, GABA content in Spirulina was on average 13.3 times and 1.41 times higher than in non-fermented and 24 h-fermented samples, respectively. Conversely, after 48 h of fermentation, L-Glu content was 1.34 times lower than in 24 h-fermented samples on average. Both GABA and L-Glu concentration values showed, respectively, negative very strong and strong correlations with Spirulina pH (r = −0.951, *p* ≤ 0.0001 and r = −0.807, *p* = 0.009, respectively). 

The production of GABA by microorganisms depends on many factors, and one of them is the pH, which affects the biosynthesis of GABA by microorganisms [12]. It was reported that *Lactiplantibacillus plantarum* C48 in cheese produced a high concentration of GABA (289–391 mg/kg) at a wide range of pH values (from 4.68 to 5.70) [21]. However, GABA synthesis was reduced at a pH value of 8 [40]. It was suggested that timely adjustment of fermentable substrate pH should be conducted to optimal values so as to obtain the highest GABA yield [16,21]. Additionally, the high-efficiency glutamate conversion to GABA requires an appropriate temperature [16]. It was reported that an optimum temperature for GABA production using *Lentilactobacillus buchneri* in MRS broth was 30 °C [16]. Nevertheless, optimal temperature is strain dependent; *Levilactobacillus brevis* produced 92% GABA at 40 °C [41], *Streptococcus salivarius* subsp. *thermophilus* (or simply *Streptococcus thermophilus*) at 37 °C [42], *Lacticaseibacillus paracasei* NFRI 7415 at 37 °C [43]. In this study, a temperature of 30 °C was employed for Spirulina fermentation with *Lactiplantibacillus plantarum* No. 122 strain, as this is the optimal growth temperature [44].

Moreover, duration of fermentation also plays an important role in GABA production [10,27]. In this study, the test of between-subjects effects showed that the duration of fermentation was a statistically significant factor on the production of both GABA and L-Glu (*p* ≤ 0.001).

L-Glu is a multifunctional amino acid involved in intermediary metabolism, taste perception, and excitatory neurotransmission [45]. It is also a specific precursor for bioactive molecules such as γ-amino butyric acid (GABA) and glutathione [46].

Studies concerning L-Glu production by LAB are scarce [47]. It was established that some LAB can produce glutamic acid [46], and the presence of glutamic acid dehydrogenase (or glutamate dehydrogenase, GDH) gene in LAB was also reported [48].

Screening of various LAB capable of producing glutamic acid may be used to explore new avenues for the development of functional foods rich in GABA [46]. It was reported that cytoplasmic glutamate dehydrogenase occurs in *Lactiplantibacillus plantarum* [49]. However, another study reported that the intra-cellular concentration of L-Glu often de-creases after 48 h to an extent, owing to its secretion into the extra-cellular medium [50]. This finding, along with the reported localization of glutamic acid dehydrogenase in cytoplasm, suggests that glutamic acid was synthesised in the cytoplasm of *Lactiplantibacillus plantarum* and then secreted into the culture medium [46]. The results of the current study showed that *Lactiplantibacillus plantarum* No. 122 strain is able to produce L-Glu, but the L-Glu concentration decreased after 48 h of fermentation, and this can be explained by a decrease in LAB conversion capacities as well as by L-Glu conversion to GABA, which is demonstrated by the fact that GABA content after 48 h of fermentation was higher than in the 24 h-fermented samples.

The fatty acid (FA) profile of Spirulina samples is presented in Table 4. The main FA in Spirulina were palmitic (C16:0), linoleic (C18:2), and gamma linolenic (C18:3ɣ) acids. Additionally, in 48 h-fermented samples, alfa-linolenic acid (C18:3α) was established (0.605% of total fat content). Comparing non-treated samples with 24 h-fermented ones, different tendencies were observed, and fermentation increased C16:0, stearic acid (C18:0), and C18:3ɣ contents (on average 3.88, 19.1, and 9.21%, respectively) and reduced *cis*, *trans*-9- oleic acid (C18:1 *cis*, *trans*) and C18:2 contents (on average 4.53 and 16.2%, respectively) in Spirulina. Nevertheless, after 48 h of fermentation, C16:0 content in Spirulina decreased in comparison with 24 h-fermented samples—the opposite is true of C18:0 and C18:3ɣ, the contents of which after 48 h of fermentation were higher in comparison with 24 h-fermented samples (on average 33.7 and 7.83%, respectively). Despite palmitoleic (C16:1) contents in non-treated and 24 h-fermented samples being similar, its content decreased, on average, by 5.05% after 48 h of fermentation. Saturated FA were the main form of FA in Spirulina, with the highest content obtained in 24 h-fermented samples (56.8% of total fat content). Fermented samples (24 and 48 h) showed, on average, 1.85% lower monounsaturated FA content; however, significant differences between polyunsaturated FA were not established. Fermentation showed a trend of reducing omega-6 and increasing omega-3 content in Spirulina.

The lipid concentration of Spirulina can vary from ca. 5 to 10% (of dry weight) [51]. Ljubic, Safafar, Holdt, and Jacobsen, [52] stated that the most common lipids in Spirulina are γ-linolenic acid (18:3, n-6, from the omega-6 family) and palmitic acid (16:0).

The long-chain FA (palmitic acid and gamma-linoleic acid) are predominant in Spirulina [53,54]. However, it was verified that palmitic, oleic, and linoleic acid contents in Spirulina can be higher (46, 8, and 12%, respectively) [55]. Gamma-linoleic acid is the most significant polyunsaturated FA [56,57]. In addition to the FA profile of non-treated Spirulina, it was reported that 6 days of solid-state fermentation (SSF) with the fungus *Aspergillus niger*, *Spirulina* spp. led to a linoleic acid content of 60.63% (of total fat content), which was much higher than that obtained by SSF with *Lactiplantibacillus plantarum* (16.93%). The changes in FA profile are explained by the reduction of substrate concentration during the fermentation process; the nutrients were used for the microbial growth and production of secondary metabolites [57]. Omega-6 constitutes the majority of the total Spirulina FA [58,59]. Moreover, Spirulina contains a significant amount of palmitic acid (16:0), which represents more than 25% from the total fat content [51]. Spirulina has been recommended as a food supplement in cases of essential FA deficiency [60]. In Spirulina, PUFA content represents 30% of the total fat content [61]. Another study reported that the FA profile of Spirulina contains sapienic (2.25%), linoleic (16.7%), and *γ*-linolenic (14%) acids [62]. According to Al-Dhabi and Valan Arasu [63], the FA of Spirulina encompasses myristic, heptadecanoic, stearic, oleic, palmitoleic, omega-3, omega-6, linoleic, and palmitic acids. According to Al-Dhabi and Valan Arasu [63], myristic, stearic, and eicosadienoic acids were the predominant saturated FA in Spirulina. Spirulina is the only food source that contains large amounts of essential FA, especially *γ*-linolenic acid. Finally, it is important to mention that the FA profile of Spirulina biomass can be modified by applying fermentation: by changing technological parameters (duration of fermentation, strain, etc.), the FA profile changes, so the FA profile in the end product should be evaluated.

### 3.2. Parameters of Nutraceutical Chewing Candies

Images of produced nutraceutical chewing candies are depicted in Table 5. Spirulina has wide food applications including juice smoothies, salad dressing, breakfast cereals, etc. [20]. In addition to the higher nutritional value and therapeutic benefits, it was reported that supplementation of Spirulina in the foods leads to better texture and colour parameters of the products [64]. Moreover, in this study, before the application of fermented Spirulina for chewing candy preparation, sugar, in gummies recipe, was changed to xylitol, and, because the differences in products overall acceptability were not found (both recipes were evaluated, on average by 8.7 scores), further samples prepared with xylitol were analysed.

**Figure 2:**
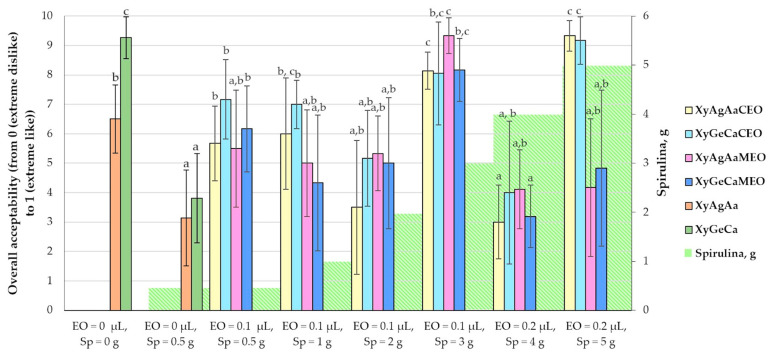
Mean values and standard deviations of overall acceptability of nutraceutical chewing candies. Xy—Xylitol; Ag—Agar; Ge—Gelatine; Aa—Ascorbic acid; Ca—Citric acid; Sp—fermented Spirulina (*Arthrospira platensis*); CEO—*Citrus paradise* essential oil; MEO—*Mentha spicata* essential oil. Data are represented as means (n = 30) ± standard errors. a–c—mean values denoted with different letters indicate significantly different values between the different samples (*p* ≤ 0.05).

Overall acceptability of nutraceutical chewing candies is shown in Figure 2. It can be observed that in comparison with the gummies prepared without Spirulina, microalgae addition at low concentration (0.5 g) led to a significantly lower overall acceptability (in comparison with samples without Spirulina in which, on average, 55.9% lower overall acceptability was attained). However, the addition of essential oils (both tested essential oils at concentrations of 0.1 μL) significantly increased the overall acceptability of the samples prepared with gelatine as a texture forming agent. Furthermore, when increasing Spirulina content in nutraceuticals (up to 1.0 g), samples prepared with *Citrus paradise* essential oil showed slightly higher overall acceptability in comparison with those prepared with *Mentha spicata* essential oil. Similar trends in the sample group prepared with 2 g of Spirulina and 0.1 μL of essential oils were established. However, by increasing the content of Spirulina to 3 g, the overall acceptability of the samples was increased. Moreover, by increasing Spirulina content to 4 g, the essential oil concentration in recipe increased to 0.2 μL, leading to lower acceptability scores. Moreover, by increasing Spirulina to 5 g, very high overall acceptability was attained in samples supplemented with *Citrus paradise* essential oil (in comparison with samples prepared with *Mentha spicata* essential oil, they were, on average, 2.06 times higher). Incorporation of microalgae in foods can lead to various forms of sensory acceptability due to the different processing and balance of the ingredients. Because of changes in colour, flavour, and texture, product acceptability can be reduced when microalgae concentration rises [65]. Paternina et al. [66] found that gummy candies with Spirulina at concentrations of 1, 3, and 5% received scores for overall acceptability between 7.09 and 7.22. In the study of Tiepo et al. [65], the overall impression of ice cream with Spirulina was lower than the control ice cream. According to Lucas et al. [67], the overall acceptance of extruded snacks with Spirulina was expressed as “like moderately” and “like very much”.

**Figure 3:**
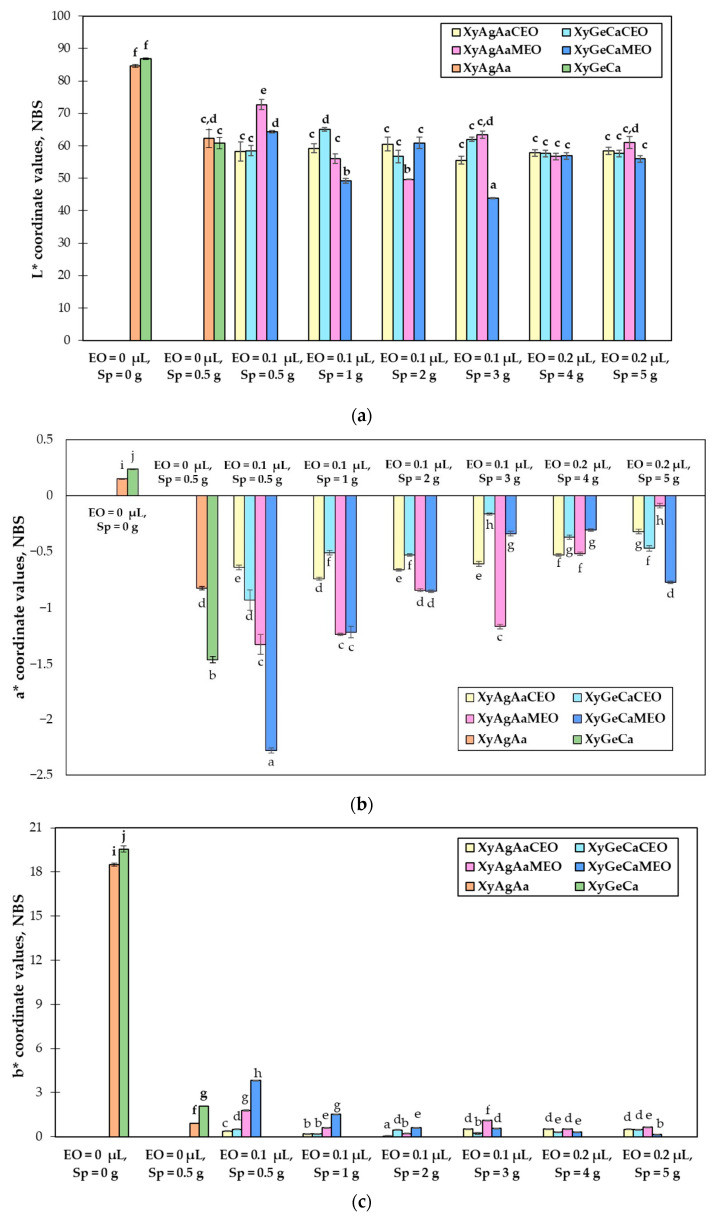
Mean values and standard deviations of colour coordinates of nutraceutical chewing candies: (**a**) lightness; (**b**) redness; and (**c**) yellowness. Xy—Xylitol; Ag—Agar; Ge—Gelatine; Aa—Ascorbic acid; Ca—Citric acid; Sp—fermented Spirulina (*Arthrospira platensis*); CEO—*Citrus paradise* essential oil; MEO—*Mentha spicata* essential oil. L*—lightness; a*—redness or −a*—greenness; b*—yellowness or −b*—blueness; NBS—National Bureau of Standards units. Data are represented as means (n = 6) ± standard errors. a–j—mean values denoted with different letters indicate significantly different values between the different samples with different EO and spirulina concentrations (*p* ≤ 0.05).

Colour coordinates of nutraceutical chewing candies are shown in Figure 3a–c. The highest values of the lightness (L*) coordinate were obtained in XyAgAa and XyGeGa samples, prepared without the addition of Spirulina (84.7 and 86.9 NBS, respectively) (Figure 3a). All samples containing Spirulina had lower values of L* coordinate, with the lowest (43.9 NBS) being obtained in the sample XyGeCaSp_3_MEO_0.1_. Despite different Spirulina contents and *Citrus paradise* oil concentrations in all XyAgAaCEO and XyGeCaCEO samples, values of L* coordinate were similar. The lightness of the previously mentioned chewing candies was also similar to the following samples: XyAgAaSp_1_MEO_0.1_, XyGeCaSp_1_MEO_0.1_, XyAgAaSp_3_MEO_0.1_, XyAgAaSp_4_MEO_0.2_, XyGeCaSp_4_MEO_0.2_, XyAgAaSp_5_MEO_0.2_, and XyGeCaSp_5_MEO_0.2_.

Chewing candies without Spirulina had positive values of a* coordinate in the range of 0.151–0.238 NBS (Figure 3b). The values of b* coordinate for chewing candies without Spirulina were in the range of 18.5–19.6 NBS, and these values were the highest when compared to other tested samples (Figure 3c). The addition of Spirulina significantly decreased the yellowness (b*) and increased the greenness (−a*) of all nutraceutical chewing candies. However, clearer trends in relation to the different microalgae content could not be drawn. It was observed that, in most cases, XyAgAaMEO and XyGeCaMEO samples with Spirulina had lower values of a* coordinate, compared to XyAgAaCEO and XyGeCaCEO with Spirulina, respectively. The lowest value of a* (−2.28 NBS) was found for XyGeCa samples prepared with 0.5 g of Spirulina and 0.1 μL of *Mentha spicata* essential oil. The lowest value of b* (0.034 NBS) was found in XyAgAa samples prepared with 2 g of Spirulina and 0.1 μL of *Citrus paradise* essential oil.

Due to the presence of chlorophylls and carotenoids, Spirulina has demonstrated strong pigment potential, making it a suitable alternative to synthetic colourants [66]. Moreover, these pigments possess antioxidant, antiviral, anti-mutative, and antitumor properties [2]. The dark green colour of the samples is due the chlorophyll, which is predominant in spirulina powders, whereas redness and blueness may indicate the presence of carotenoids and C-phycocyanin [68]. Similar to our results, Paternina et al. [66] also reported lower values of L* and a* coordinates in gummy candies enriched with Spirulina when compared to the control.

**Figure 4:**
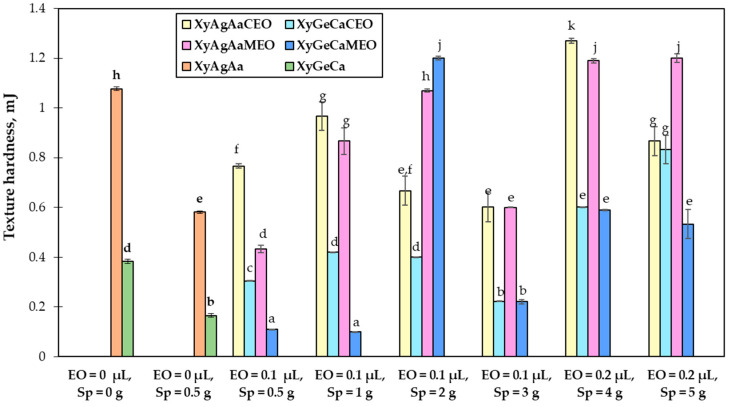
Mean values and standard deviations of texture hardness of nutraceutical chewing candies. Xy—Xylitol; Ag—Agar; Ge—Gelatine; Aa—Ascorbic acid; Ca—Citric acid; Sp—fermented Spirulina (*Arthrospira platensis*); CEO—*Citrus paradise* essential oil; MEO—*Mentha spicata* essential oil. Data are represented as means (n = 6) ± standard errors. a–k—mean values denoted with different letters indicate significantly different values between samples (*p* ≤ 0.05).

Texture hardness of nutraceutical chewing candies is shown in Figure 4. The hardness of XyAgAa and XyGeCa samples prepared with 0.5 g of Spirulina was significantly lower (on average 46% and 56.8%, respectively) when compared to controls. Addition of 0.1 μL of *Citrus paradise* essential oil increased the hardness of XyAgAa and XyGeCa samples with 0.5 g of Spirulina. On the contrary, addition of 0.1 μL of *Mentha spicata* essential oil decreased the hardness of XyAgAa and XyGeCa samples with 0.5 g of Spirulina. Addition of 1 g of Spirulina significantly increased the hardness of XyAgAaSp_1_CEO_0.1_, XyAgAaSp_1_MEO_0.1_, and XyGeCaSp_1_CEO_0.1_, compared to samples with 0.5 g of Spirulina. However, changes in hardness of samples with higher content of Spirulina and essential oils were uneven. XyGeCaSp_2_MEO_0.1_, XyAgAaSp_4_MEO_0.2_, and XyAgAaSp_5_MEO_0.2_ shared similar hardness, which was higher than that of samples with 0.5 and 1 g of Spirulina. XyAgAaSp_2_CEO_0.1_, XyAgAaSp_3_CEO_0.1_, XyAgAaSp_3_MEO_0.1_, XyGeCaSp_4_CEO_0.2_, XyGeCaSp_4_MEO_0.2_, and XyGeCaSp_5_MEO_0.2_ had similar values of hardness, which (except for the samples with gelatine, citric acid, and *Mentha spicata* essential oil) was lower compared to samples with 0.5 and 1 g of Spirulina. The lowest hardness (0.10 mJ) was reached in XyGeCaSp_0.5_MEO_0.1_ and XyGeCaSp_1_MEO_0.1_. The highest texture hardness (1.27 mJ) was found in XyAgAa with 4 g of Spirulina and 0.1 μL of *Citrus paradise* essential oil.

The hardness changes caused by the addition of Spirulina could be explained by the reduction of the number of flexible cross-links in chewing candies and the formation of a more heterogeneous network structure [69]. It was also observed that in most cases, chewing candies with agar and ascorbic acid had higher hardness than candies with gelatine and citric acid. Such an observation can be explained by the fact that agar forms firm and clear gels at very low concentrations and these gels are stable over a wide range of temperatures, whereas a higher concentration of gelatine is required to produce firm and chewy but thermo-reversible gels [69].

Since foods can elicit specific feelings, emotions have a significant role in food choice [70]. The use of non-verbal markers such as facial expressions is a novel sensory and emotional method that aids in understanding product liking and other emotions that affect the desire to purchase [71]. Since all samples with 3 g of Spirulina and samples with 5 g of Spirulina and 0.2 μL of *Citrus paradise* essential oil received the highest scores of overall acceptability, the intensity of emotions induced in panellists by these samples was also analysed. The elicited emotions and their intensity for tasted nutraceutical chewing candies are tabulated in Table 6. According to obtained results, the intensities of facial expressions “happy”, “sad” “angry”, “surprised”, and “disgusted” (except “neutral” and “scared”) were significantly influenced by different chewing candy types (p ≤ 0.05). The highest intensity (0.052) of “happy”, which is a positive emotion, was produced by XyAgAa chewing candy with 3 g of Spirulina and 0.1 μL of *Mentha spicata* essential oil. Tasting of samples XyGeCaSp_3.0_MEO_0.1_ and XyGeCaSp_5.0_CEO_0.2_ elicited the lowest intensity of this positive emotion. The highest facial expression “surprised”, which can be indicative of both positive and negative emotion, was expressed by XyAgAaSp_5.0_CEO_0.2_ and XyAgAaSp_3.0_CEO_0.1_. The latter sample of chewing candy elicited the highest intensities of such negative emotions as “sad” and “angry “. Intensities of these emotions were also similar with those induced in XyGeCaSp_3.0_CEO_0.1_ and XyGeCaSp_3.0_MEO_0.1_, respectively. Tasting of XyAgAa with 5 g of Spirulina and 0.2 μL of *Citrus paradise* essential oil Sp_5.0_CEO_0.2_ elicited the highest intensities of “disgusted” and “contempt”. All XyAgAa and XyGeCa samples with 3 g of Spirulina and 0.1 μL of essential oils induced similar intensities of “contempt”, which were the lowest among all samples.

To the best of the authors’ knowledge, there are no scientific records on the effect of Spirulina on the emotional profile of nutraceutical chewing candies. Only Moss et al. [72] evaluated panellists’ emotional responses towards a variety of pictures of foods with seaweed using the CATA variant of EsSense25 Profile. He found that pictures with seaweed containing bread evoked positive emotions, while beef burger, sausage, and yogurt elicited such negative emotions as “disgusted”.

## 4. Conclusions

Spirulina fermentation with *Lactiplantibacillus plantarum* No. 122 strain led to changes in colour coordinates, and a negative moderate correlation between pH and b* coordinate values of the samples was found (r = −0.775). Fermentation of Spirulina significantly increased the contents of L-glutamic and gamma-aminobutyric acids, and its duration had a statistically significant impact on the content of these acids Spirulina. Fatty acid profile was also affected by fermentation The amount of polyunsaturated FA was similar between untreated (/unfermented) and fermented Spirulina samples an increase in omega-3 content after 48 h of fermentation was observed. Different sweet tasting (sugar versus xylitol), texture forming (agar versus gelatine), and sour tasting (ascorbic acid versus citric acid) ingredients, as well as essential oils (*Citrus paradise* versus *Mentha spicata*), were used for the nutraceutical gummy formulations with fermented Spirulina. Differences in overall acceptability between samples with sugar and xylitol were not disclosed; thus, further evaluation was performed with samples prepared with xylitol. Chewing candies with 3 g of fermented Spirulina, as well as with 5 g of fermented Spirulina and 0.2 µL of *Citrus paradise* essential oil, showed the highest overall acceptability. All samples containing Spirulina had lower values of the L*, b* and a*coordinates. The composition differently affected the hardness of samples, and these changes were uneven. Additionally, the samples showing highest acceptability were tested by using the face reading technique, which indicates the intensity of emotions induced by the tested samples in trained panellists. The intensities of facial expressions “happy”, “sad” “angry”, “surprised”, and “disgusted” (excepting “neutral” and “scared”) were significantly influenced by the different chewing candy compositions (*p* ≤ 0.05). The highest intensity (0.052) of “happy” emotion was provoked by samples (with agar and ascorbic acid) containing 3 g of fermented Spirulina and 0.1 µL of *Mentha spicata* essential oil. Finally, fermented Spirulina together with *Citrus paradise* and *Mentha spicata* essential oils could be used to produce nutraceutical chewing candies (gummies) with high overall acceptability and improved functional value.

## Figures and Tables

**Table 1:** Nutraceuticals in chewing candy (gummy) formulas.

Gummy Candies	Sugar	Xylitol	Agar	Gelatine	Ascorbic Acid	Citric Acid	Spiru-Lina	Water	*C. paradise* EO	*M. spicata* EO
	g							mL	μL	
CSuAgAa	17	-	4.6	-	0.9	-	-	20	-	-
CSuGeCa	17	-	-	8.5	-	0.7	-	20	-	-
CXyAgAa	-	17	4.6	-	0.9	-	-	20	-	-
CXyGeCa	-	17	-	8.5	-	0.7	-	20	-	-
XyAgAaSp_0.5_	-	17	4.6	-	0.9	-	0.5	20	-	-
XyGeCaSp_0.5_	-	17	-	8.5	-	0.7	0.5	20	-	-
XyAgAaSp_0.5_CEO_0.1_	-	17	4.6	-	0.9	-	0.5	20	0.1	-
XyGeCaSp_0.5_CEO_0.1_	-	17	-	8.5	-	0.7	0.5	20	0.1	-
XyAgAaSp_0.5_MEO_0.1_	-	17	4.6	-	0.9	-	0.5	20	-	0.1
XyGeCaSp_0.5_MEO_0.1_	-	17	-	8.5	-	0.7	0.5	20	-	0.1
XyAgAa Sp_1.0_CEO_0.1_	-	17	4.6	-	0.9	-	1.0	20	0.1	-
XyGeCa Sp_1.0_CEO_0.1_	-	17	-	8.5	-	0.7	1.0	20	0.1	-
XyAgAa Sp_1.0_MEO_0.1_	-	17	4.6	-	0.9	-	1.0	20	-	0.1
XyGeCa Sp_1.0_MEO_0.1_	-	17	-	8.5	-	0.7	1.0	20	-	0.1
XyAgAa Sp_2.0_CEO_0.1_	-	17	4.6	-	0.9	-	2.0	20	0.1	-
XyGeCa Sp_2.0_CEO_0.1_	-	17	-	8.5	-	0.7	2.0	20	0.1	-
XyAgAa Sp_2.0_MEO_0.1_	-	17	4.6	-	0.9	-	2.0	20	-	0.1
XyGeCa Sp_2.0_MEO_0.1_	-	17	-	8.5	-	0.7	2.0	20	-	0.1
XyAgAa Sp_3.0_CEO_0.1_	-	17	4.6	-	0.9	-	3.0	20	0.1	-
XyGeCa Sp_3.0_CEO_0.1_	-	17	-	8.5	-	0.7	3.0	20	0.1	-
XyAgAa Sp_3.0_MEO_0.1_	-	17	4.6	-	0.9	-	3.0	20	-	0.1
XyGeCa Sp_3.0_MEO_0.1_	-	17	-	8.5	-	0.7	3.0	20	-	0.1
XyAgAa Sp_4.0_CEO_0.2_	-	17	4.6	-	0.9	-	4.0	20	0.2	-
XyGeCa Sp_4.0_CEO_0.2_	-	17	-	8.5	-	0.7	4.0	20	0.2	-
XyAgAa Sp_4.0_MEO_0.2_	-	17	4.6	-	0.9	-	4.0	20	-	0.2
XyGeCa Sp_4.0_MEO_0.2_	-	17	-	8.5	-	0.7	4.0	20	-	0.2
XyAgAa Sp_5.0_CEO_0.2_	-	17	4.6	-	0.9	-	5.0	20	0.2	-
XyGeCa Sp_5.0_CEO_0.2_	-	17	-	8.5	-	0.7	5.0	20	0.2	-
XyAgAa Sp_5.0_MEO_0.2_	-	17	4.6	-	0.9	-	5.0	20		0.1
XyGeCa Sp_5.0_MEO_0.2_	-	17	-	8.5	-	0.7	5.0	20		0.1

C—control; Su—sugar; Xy—xylitol; Ag—agar; Ge—gelatine; Aa—ascorbic acid; Ca—citric acid; Sp—Spirulina (*Arthrospira platensis*); EO—essential oil; CEO—*Citrus paradise* essential oil; MEO—*Mentha spicata* essential oil.

**Table 2:** Mean values and standard deviations of pH and colour coordinates (L*, a* and b*) in Spirulina samples.

Spirulina Samples	Fermentation Time, h	Colour Coordinates, NBS			pH
		L*	a*	b*	
Control	0	22.5 ± 0.1 a	0.440 ± 0.006 b	1.55 ± 0.02 a	6.33 ± 0.03 b
*Lactiplantibacillus plantarum* No. 122	24	24.5 ± 0.4 b	0.052 ± 0.001 a	2.07 ± 0.06 c	4.81 ± 0.02 a
	48	22.4 ± 0.2 a	0.480 ± 0.003 c	1.75 ± 0.01 b	4.79 ± 0.02 a

Control—Spirulina powder and water mixture, 1:2 (*w*/*w*); L*—lightness; a*—redness; b*—yellowness; NBS—National Bureau of Standards units. Data are represented as means (n = 6) ± standard errors. a–c—mean values denoted with different letters indicate significantly different values between the different samples (*p* ≤ 0.05).

**Table 3:** Mean values and standard deviations of gamma-aminobutyric acid (GABA) and L-glutamic acid (L-Glu) concentrations in Spirulina samples.

Spirulina Samples	Fermentation Duration, h	Gamma-Aminobutyric Acid, mg/kg	L-Glutamic Acid, mg/kg
Control	0	17.2 ± 0.231 a	2296 ± 11.3 a
*Lactiplantibacillus plantarum* No. 122	24	161.7 ± 8.52 b	4062 ± 10.0 c
	48	228.6 ± 9.01 c	3033 ± 10.8 b

Control—Spirulina powder and water mixture, 1:2 (*w*/*w*). Data are represented as means (n = 6) ± standard errors. a–c—mean values denoted with different letters indicate significantly different values between the different samples (*p* ≤ 0.05).

**Table 4:** Mean values and standard deviations of fatty acids (FA) in Spirulina samples.

Spirulina Samples	Fermentation Duration, h	Fatty Acid Content, % from Total Fat Content						
		C16:0	C16:1	C18:0	C18:1 *cis,* *trans*	C18:2	C18:3 γ	C18:3 α
Control	0	51.6 ± 0.5 a	3.73 ± 0.07 b	2.67 ± 0.02 a	3.31 ± 0.01 b	23.5 ± 0.20 c	15.2 ± 0.30 a	nd
*Lactiplantibacillus plantarum* No. 122	24	53.6 ± 0.2 b	3.76 ± 0.03 b	3.18 ± 0.01 b	3.16 ± 0.02 a	19.7 ± 0.10 b	16.6 ± 0.10 b	nd
	48	52.1 ± 0.4 a	3.57 ± 0.02 a	4.25 ± 0.05 c	3.33 ± 0.02 b	18.2 ± 0.20 a	17.9 ± 0.20 c	0.605 ± 0.011
Classification of Fatty Acid (FA) in the Spirulina Samples.								
	Fermentation duration, h		SFA	MUFA	PUFA	Omega-3	Omega-6	Omega-9
Control	0		54.2 ± 1.20 a	7.04 ± 0.210 b	38.7 ± 1.20 a	nd	38.7 ± 1.20 b	7.04 ± 0.21 a
*Lactiplantibacillus plantarum* No. 122	24		56.8 ± 1.30 b	6.92 ± 0.200 a	36.3 ± 1.10 a	nd	36.3 ± 1.30 a	6.92 ± 0.20 a
	48		56.4 ± 1.80 b	6.90 ± 0.140 a	36.7 ± 1.00 a	0.605 ± 0.018	36.1 ± 1.00 a	6.98 ± 0.140 a

Control—Spirulina powder and water mixture, 1:2 *w*/*w*; C16:0—palmitic acid; C16:1 –palmitoleic acid; C18:0—stearic acid; C18:1 cis, trans—cis, trans-9- oleic acid; C18:2 –linoleic acid; C18:3ɣ—gamma-linolenic acid; C18:3α—alfa-linolenic acid; SFA—saturated fatty acids; MUFA—monounsaturated fatty acids; PUFA—polyunsaturated fatty acids; omega 3—ω-3 fatty acids; omega 6—ω-6 fatty acids; omega 9—ω-9 fatty acids; nd—not determined. Data are represented as means (n = 6) ± standard errors. a–c—mean values denoted with different letters indicate significantly different values between the different samples (*p* ≤ 0.05).

**Table 5:** Images of nutraceutical chewing candies (gummies).

CSuAgAa	CSuGeCa	CXyAgAa	CXyGeCa	XyAgAa Sp_3.0_CEO_0.1_
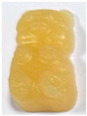	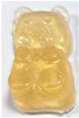	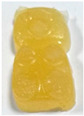	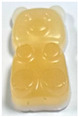	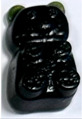
XyGeCa Sp_3.0_CEO_0.1_	XyAgAa Sp_3.0_MEO_0.1_	XyGeCa Sp_3.0_MEO_0.1_	XyAgAa Sp_5.0_CEO_0.2_	XyGeCa Sp_5.0_CEO_0.2_
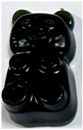	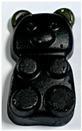	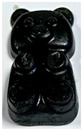	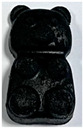	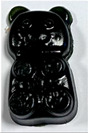

C—control; Su—Sugar; Xy—Xylitol; Ag—Agar; Ge—Gelatine; Aa—Ascorbic acid; Ca—Citric acid; Sp—fermented Spirulina (*Arthrospira platensis*); CEO—*Citrus paradise* essential oil; MEO—*Mentha spicata* essential oil.

**Table 6:** Mean values and standard deviations of intensity of emotions induced in in panellists by nutraceutical chewing candies.

Chewing Candies	Intensity of Emotions Induced in Panellists							
	Neutral	Happy	Sad	Angry	Surprised	Scared	Disgusted	Contempt
XyAgAa Sp_3.0_CEO_0.1_	0.839 ± 0.056 a	0.022 ± 0.003 c	0.029 ± 0.003 c	0.021 ± 0.002 d	0.063 ± 0.006 c	0.002 ± 0.001 a	0.008 ± 0.001 b	0.001 ± 0.001 a
XyGeCa Sp_3.0_CEO_0.1_	0.845 ± 0.061 a	0.036 ± 0.002 d	0.024 ± 0.003 c	0.010 ± 0.001 b	0.012 ± 0.001 a	0.001 ± 0.001 a	0.003 ± 0.001 a	0.002 ± 0.001 a
XyAgAa Sp_3.0_MEO_0.1_	0.826 ± 0.088 a	0.052 ± 0.006 e	0.012 ± 0.002 b	0.016 ± 0.001 c	0.017 ± 0.001 a	0.001 ± 0.001 a	0.007 ± 0.002 b	0.001 ± 0.001 a
XyGeCa Sp_3.0_MEO_0.1_	0.856 ± 0.097 a	0.006 ± 0.005 a	0.012 ± 0.001 b	0.023 ± 0.002 d	0.050 ± 0.004 b	0.001 ± 0.001 a	0.006 ± 0.001 b	0.001 ± 0.002 a
XyAgAa Sp_5.0_CEO_0.2_	0.876 ± 0.077 a	0.015 ± 0.001 b	0.006 ± 0.001 a	0.004 ± 0.001 a	0.056 ± 0.006 bc	0.001 ± 0.001 a	0.012 ± 0.001 c	0.024 ± 0.003 c
XyGeCa Sp_5.0_CEO_0.2_	0.909 ± 0.048 a	0.008 ± 0.001 a	0.011 ± 0.001 b	0.004 ± 0.001 a	0.049 ± 0.005 b	0.003 ± 0.001 a	0.004 ± 0.001 a	0.010 ± 0.001 b

C—control; Xy—Xylitol; Ag—Agar; Ge—Gelatine; Aa—Ascorbic acid; Ca—Citric acid; Sp—fermented Spirulina (*Arthrospira platensis*); CEO—*Citrus paradise* essential oil; MEO—*Mentha spicata* essential oil. Data are represented as means (n = 30) ± standard errors. a–e—mean values denoted with different letters indicate significantly different values between the different samples (*p* ≤ 0.05).

## Data Availability

The data are available from the corresponding author upon reasonable request. The data presented in this study are openly available in this manuscript.
